# Rapid containment of a hospital outbreak of Ralstonia mannitolilytica bloodstream infection linked to contaminated pre-filled 0.9% saline vials

**DOI:** 10.1017/ash.2026.10815

**Published:** 2026-09-01

**Authors:** Ibrahim Al-Busaidi, Hawra Al-Lawati, Turkiya Al-Siyabi, Asma Al-Balushi, Divya Deodhar, Ghada Al-Rawahi, Azza Al-Rashdi, Iman Al-Balushi, Abdullah Al-Rawahi, Intisar Al-Yahmadi, Mansour Al-Jabri, Hazza Al-Yahyai, Amani Al-Siyabi, Manawr Al-Quraini, Maya Al-Hattali, Maryam Al-Shuaili, Zeid Al-Hinai, Laila Al-Yazidi, Abdulhakim Al-Rawas, Thuraya Al-Busaidi, Salama Al-Riyami

**Affiliations:** 1 Sultan Qaboos University Hospital, Oman; 2 CPHL, Ministry of Health, Oman; 3 Al Buraimi Hospital, Ministry of Health, Oman

## Abstract

**Background:**

*Ralstonia* species are environmental Gram-negative bacilli increasingly linked to healthcare-associated bloodstream infection (BSI) outbreaks from contaminated medical solutions. We describe an outbreak of *Ralstonia mannitolilytica* BSI in which the source was identified and withdrawn within 24 hours.

**Methods:**

Following an unusual cluster of *R. mannitolilytica* blood cultures on 29 September 2024 at a 700-bed tertiary-care hospital in Oman, we conducted a multidisciplinary investigation including chart review, hospitalwide surveillance, environmental and product sampling, antimicrobial susceptibility testing, and pulsed-field gel electrophoresis (PFGE) of clinical and product isolates.

**Results:**

Between 29 September and 8 October 2024, 21 patients met the case definition (median age 9 yr; 62% pediatric; 62% male; 95% immunocompromised; 86% with central venous access). Newly introduced pre-filled 10 mL 0.9% sodium chloride vials from a single manufacturer yielded *R. mannitolilytica* with concordant clinical and product antimicrobial susceptibility profiles. PFGE of 18 clinical isolates and one product isolate demonstrated 100% genetic relatedness, confirming a single-clone outbreak. The implicated product was withdrawn within 24 hours of cluster recognition. Thirteen of 21 patients had their central catheters removed; all received intravenous trimethoprim–sulfamethoxazole. Two patients experienced bacteremia recurrence after initial line retention; no outbreak-related deaths occurred.

**Conclusions:**

Prefilled saline vials can serve as the source of clonal *R. mannitolilytica* BSI outbreaks in immunocompromised patients. Laboratory-driven cluster detection, multidisciplinary coordination, and rapid product suspension enabled source identification within 24 hours and limited outbreak size and severity. Vigilance after introduction of new pharmaceutical products and prompt catheter removal in catheter-associated *Ralstonia* BSI are essential.

## Introduction

*Ralstonia* species are aerobic, non-fermentative Gram-negative bacilli that have emerged as opportunistic pathogens capable of causing nosocomial invasive infections—including central line–associated bloodstream infection (CLABSI) and meningitis—predominantly in immunocompromised patients.^1,2^ *Ralstonia pickettii* has been the most commonly reported species, but *Ralstonia mannitolilytica* and *Ralstonia insidiosa* are increasingly recognized as outbreak pathogens.^3–6^ These organisms cause outbreaks through contaminated water-based products, including intravenous fluids, heparinized syringes, hemodialysis water, antiseptics, and saline and medication vials.^2,7–11^ Their tolerance of low nutrient concentrations, growth across a wide temperature range (15–42°C), small cell size permitting passage through 0.2 µm sterilizing filters, and biofilm formation enhance product contamination and nosocomial risk.^1,3,4^

*Ralstonia* species exhibit intrinsic resistance to many β-lactams and aminoglycosides, while trimethoprim–sulfamethoxazole and fluoroquinolones generally remain active.^12,13^ Reported case-fatality rates among *Ralstonia* BSI cohorts have been substantial—up to 38.8% for *R. mannitolilytica* and 60% for *R. pickettii*.^14,15^ Timely source identification, decisive infection-prevention action, and inter-institutional communication are therefore essential. We describe the investigation and management of a hospital outbreak of *R. mannitolilytica* BSI in which the contaminated source was identified and withdrawn within 24 hours.

## Methods

### Setting and case definition

The investigation was conducted at Sultan Qaboos University Hospital (SQUH), a 700-bed tertiary-care, University Medical City–affiliated hospital in Muscat, Oman, that serves pediatric and adult patients, including hematology–oncology and hematopoietic stem-cell transplant (HSCT) populations. A case was defined as any patient admitted to SQUH with at least one blood culture positive for *R. mannitolilytica* between 29 September and 8 October 2024.

### Outbreak detection and field investigation

On 29 September 2024, the clinical microbiology laboratory identified an unusual cluster of three *R. mannitolilytica*–positive blood cultures from different pediatric hematology–oncology patients on the same ward within 48 hours; the same day, four additional patients had Gram-negative bacilli grow from blood cultures, three confirmed as *Ralstonia* species that day and one the following day. Microbiology immediately notified the IPC team, which initiated the investigation. Affected wards were visited, charts were reviewed, and adherence to standard IPC practices was reviewed and reinforced. Active hospitalwide surveillance for further *Ralstonia* BSI was implemented.

### Environmental and product sampling

Because all initial cases had central venous access and were receiving intravenous therapy, a contaminated infusate was suspected as the common source. On the day of notification, multiple intravenous solutions—including newly introduced pre-filled 10 mL 0.9% sodium chloride vials, large-volume 0.9% saline bags from different manufacturers, and a new batch of chlorhexidine antiseptic applicators—were submitted to the laboratory, together with environmental swabs from medication-preparation areas on the affected wards. Use of all suspected products was suspended hospitalwide pending testing. Intravenous solutions were cultured in BACTEC blood culture bottles and on solid media; uninoculated bottles from the same lots were incubated concurrently as a sterility check.

### Microbiological identification and susceptibility testing

Blood cultures were processed in the BACTEC FX system (Becton Dickinson). Positive bottles underwent Gram stain, identification by matrix-assisted laser desorption ionization–time-of-flight mass spectrometry (MALDI-TOF MS; Biotyper, Bruker),^16^ and subculture. Antimicrobial susceptibility testing was performed using the BD Phoenix system with gradient-diffusion confirmation when needed, and results were interpreted using Clinical and Laboratory Standards Institute (CLSI) breakpoints for non-Enterobacterales.^17^

### Molecular typing

Pulsed-field gel electrophoresis (PFGE) was performed at the national reference laboratory (Central Public Health Laboratory, Ministry of Health, Oman) on *R. mannitolilytica* isolates from 18 patients and from one contaminated saline vial, following the U.S. Centers for Disease Control and Prevention (CDC) PulseNet protocol with SpeI restriction;^18,19^ technical conditions are provided in the Supplementary Methods.

### Ethics

The investigation was conducted as part of the institutional outbreak response. The SQUH Medical Research and Ethics Committee reviewed the project; informed consent was waived because data were collected during an outbreak investigation and de-identified prior to analysis.

## Results

### Outbreak description

**Figure 1.**
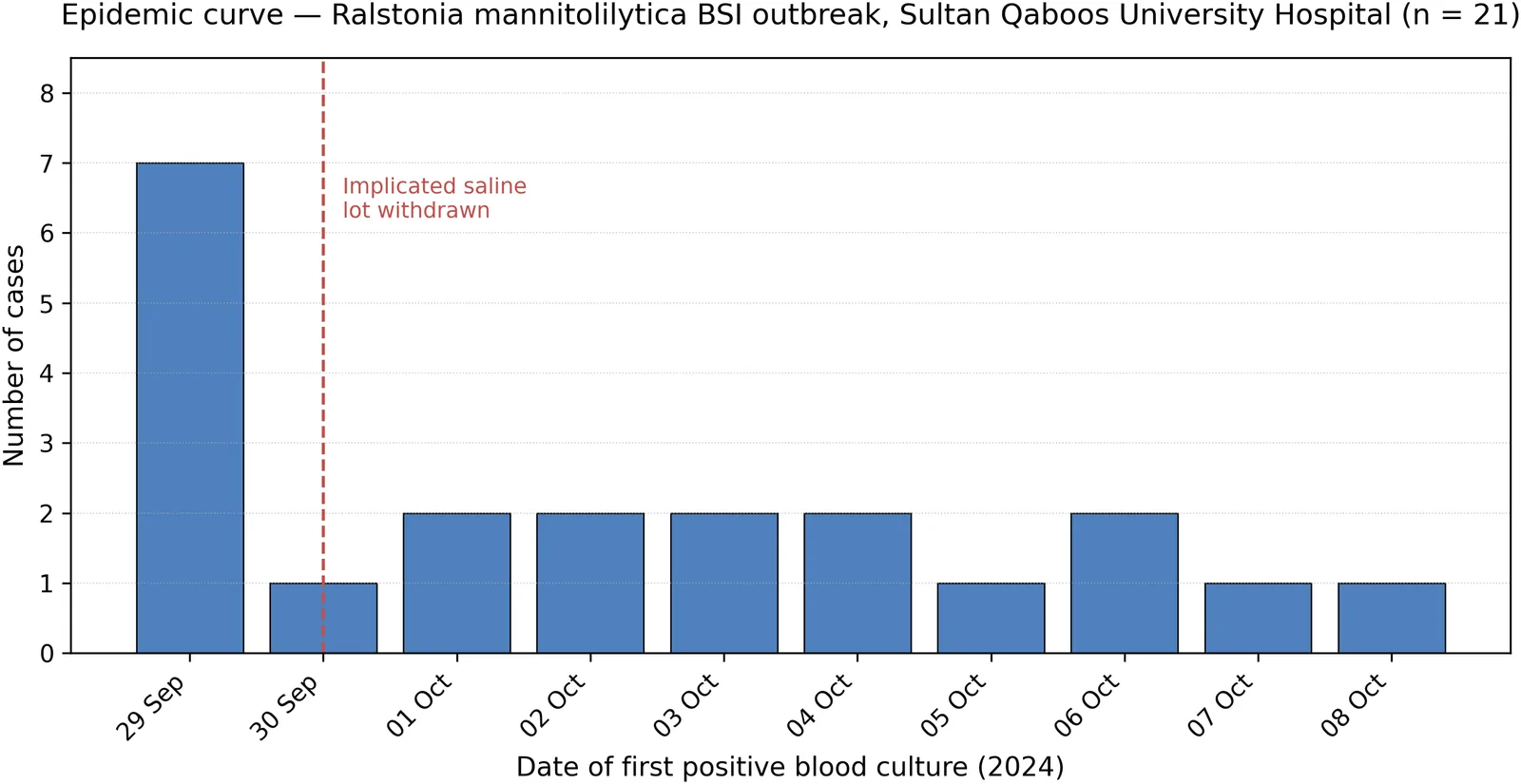
Epidemic curve of *Ralstonia mannitolilytica* bloodstream infections by date of first positive blood culture, Sultan Qaboos University Hospital, 29 September–8 October 2024 (n = 21). The vertical dashed line indicates withdrawal of the implicated pre-filled 10 mL 0.9% sodium chloride vials from clinical use within 24 hours of cluster recognition.

Between 29 September and 8 October 2024, 21 patients met the case definition (Figure 1; Supplementary Table S1). Thirteen (62%) were pediatric and 8 (38%) were adults; 13 (62%) were male; ages ranged from 6 months to 67 years. Twenty patients (95%) were immunocompromised, predominantly with hematological malignancies or postHSCT status. Eighteen (86%) had a central venous access device—CVC, Hickman catheter, port-a-cath, or peripherally inserted central catheter (PICC)—and 3 (14%) had only a peripheral intravenous line. Cases occurred across four clinical areas: the pediatric and adult hematology–oncology wards and the pediatric and adult intensive care units. The implicated pre-filled saline vials were the standard flush used for vascular-access maintenance and intravenous medication preparation and administration on all affected units; documented exposure to the implicated product was confirmed in all 21 cases (100%).

### Microbiological findings

All 21 patients had blood cultures that grew *R. mannitolilytica* on MALDI-TOF MS; in 4 patients, *R. pickettii* was co-isolated only after 48 hours of incubation. All *R. mannitolilytica* isolates were susceptible to trimethoprim–sulfamethoxazole and resistant to ceftazidime, cefepime, gentamicin, amikacin, ciprofloxacin, levofloxacin, meropenem, and colistin; all but one were non-susceptible to piperacillin–tazobactam. The 4 *R. pickettii* co-isolates were susceptible to trimethoprim–sulfamethoxazole and, except one, to piperacillin–tazobactam.

### Source identification

At least one sample from each of three lots of the implicated pre-filled 10 mL 0.9% sodium chloride vials was cultured, but only one lot (Sodium Chloride Injection B.P. 0.9% w/v; M/s Bioreva, Republic of Ireland; batch no. 2724-046) was positive, yielding *R. mannitolilytica* from enrichment blood culture bottles after 24 hours and from direct culture on solid media after 48 hours. The newly introduced brand of 0.9% saline vials had been received and started in clinical use on the affected units 5 days before the cluster was detected. Antimicrobial susceptibility testing of the product isolate matched the clinical isolate profile (susceptible only to trimethoprim–sulfamethoxazole). All other tested intravenous solutions, chlorhexidine applicators, environmental swabs, and the blank uninoculated blood culture bottles (sterility check) were negative. PFGE of *R. mannitolilytica* isolates from 18 patients and from one contaminated saline vial demonstrated 100% band-pattern similarity, indicating a single outbreak clone (Supplementary Figure S2). Within 24 hours of cluster recognition, all units of the implicated saline lot were withdrawn from clinical use hospitalwide and the supplier and the Ministry of Health Drug Safety Center (DSC) were notified; a nationwide batch recall was announced by the DSC shortly after notification.

### Clinical management and outcomes

All 21 patients received intravenous trimethoprim–sulfamethoxazole as definitive therapy after susceptibility results were available; empirical regimens at presentation are summarized in Supplementary Table S1. Central venous catheters were removed in 13 of 21 patients (62%); one patient with a port-a-cath was managed with antimicrobial lock therapy and line retention. Two patients with initial line retention experienced relapse of *R. mannitolilytica* bacteremia 1 week and 5 weeks after completion of antibiotics, respectively; both responded after device removal and a second course of trimethoprim–sulfamethoxazole. Three patients required intensive-care admission for sepsis or septic shock (one requiring non-invasive ventilation and inotropic support); transthoracic echocardiography in two patients showed no vegetations. No outbreak-related deaths occurred, and all patients recovered.

## Discussion

We report a hospital outbreak of 21 cases of *R. mannitolilytica* bloodstream infection traced to contaminated pre-filled 10 mL 0.9% sodium chloride vials in immunocompromised patients with central venous access. Laboratory-driven cluster detection, immediate IPC notification, and a coordinated multidisciplinary response—including microbiology, infectious diseases, IPC, pharmacy, nursing, and hospital administration—enabled identification and removal of the contaminated product within 24 hours of cluster recognition.

Despite hospitalwide distribution of the implicated product, clinical infections occurred almost exclusively in immunocompromised patients with central catheters, consistent with combined host susceptibility and intravascular biofilm-mediated pathogenesis.^20–22^

*R. pickettii* was co-isolated in 4 of 21 patients, in each case only after 48 hours of incubation and always together with *R. mannitolilytica*. *R. pickettii* was not recovered from the implicated saline vials or from any other product or environmental sample, and the outbreak clone defined by PFGE was *R. mannitolilytica*. The two species commonly co-occur in water-based reservoirs, and low-level co-contamination or recovery of a slow-growing commensal cannot be excluded; however, the clinical significance of the co-isolates is uncertain, and they did not alter management because all isolates were susceptible to trimethoprim–sulfamethoxazole.

A notable feature of this investigation was the speed of source identification. Nonetheless, seven bacteremias occurred within the first 48 hours before the cluster was acted upon, and saline-associated *Ralstonia* outbreaks had already been reported in several jurisdictions. Because *Ralstonia* is a rare and characteristically product-associated pathogen, even a single case of *Ralstonia* spp. bacteremia—particularly in a patient receiving sterile infusates—should prompt immediate investigation and a low threshold for suspending potentially implicated medications or solutions pending results. Microbiology recognition of an unusual same-day cluster, immediate IPC engagement, and protective suspension of the suspected lot before microbiological confirmation appear to have limited outbreak size and severity.

Several late-onset infections occurred more than one week after product withdrawal, all in immunocompromised patients with intravascular devices. Both relapses occurred after initial line retention and resolved only after device removal—consistent with, though, given the small numbers, not proof of, an important role for catheter removal in catheter-associated *Ralstonia* infection.

To our knowledge, this is the first reported *Ralstonia* outbreak from Oman. The investigation reinforces several operational lessons. First, introduction of a new pharmaceutical product—particularly a single-use sterile flush in widespread use across high-risk units—warrants heightened postintroduction surveillance; the implicated product, a newly introduced brand of 0.9% saline vials, had been in clinical use for only 5 days before the cluster was detected. Following this event, the hospital has implemented postintroduction surveillance with enhanced monitoring and sentinel culturing for newly introduced sterile pharmaceutical products and lots in high-risk units. Second, decisive temporary suspension of suspected products before microbiological confirmation is a high-value, low-cost intervention, with reintroduction once the product is exonerated. Where a suspected product is the sole available lot or a single-source item that cannot be withdrawn without disrupting essential care, we recommend a graded response rather than outright suspension: substituting an alternative formulation where one exists, restricting use to clinical necessity, reinforcing aseptic preparation, quarantining and sequestering the implicated lot for priority testing, and escalating immediately to the supplier and regulator to arrange expedited replacement stock or recall. Third, molecular typing (PFGE here; whole-genome sequencing where available) provides confirmatory evidence of clonality that strengthens the case for product recall and informs cross-border alerts for internationally distributed products.^2^

This investigation has limitations. PFGE was performed on 18 of 21 clinical isolates and on the single culture-positive product sample; although the concordant epidemiological, antibiogram, and molecular findings strongly support the implicated source, not every potential environmental sample underwent molecular comparison. Whole-genome sequencing was not available at the time of the investigation; consequently, while PFGE confirmed clonality within our outbreak, we could not determine whether our strain is related to those implicated in saline-associated *Ralstonia* outbreaks in other jurisdictions or exclude a shared, internationally distributed source at higher resolution. Finally, our experience reflects a single tertiary-care center, and delayed detection may occur in institutions with different surveillance systems or smaller microbiology capacity.

In conclusion, pre-filled saline vials can serve as a source of clonal *R. mannitolilytica* BSI outbreaks among immunocompromised patients. Robust laboratory surveillance, immediate multidisciplinary engagement, decisive product suspension, and timely catheter removal enabled containment within 24 hours and prevented outbreak-related mortality. Vigilance after introduction of new pharmaceutical products, a low threshold for investigating even single cases, and inter-institutional communication are essential to limit similar events.

## Supporting information

10.1017/ash.2026.10815.sm001 Al-Busaidi et al. supplementary material 1 Al-Busaidi et al. supplementary material

10.1017/ash.2026.10815.sm002 Al-Busaidi et al. supplementary material 2 Al-Busaidi et al. supplementary material

10.1017/ash.2026.10815.sm003 Al-Busaidi et al. supplementary material 3 Al-Busaidi et al. supplementary material

10.1017/ash.2026.10815.sm004 Al-Busaidi et al. supplementary material 4 Al-Busaidi et al. supplementary material

10.1017/ash.2026.10815.sm005 Al-Busaidi et al. supplementary material 5 Al-Busaidi et al. supplementary material

## Supplementary material

The supplementary material for this article can be found at https://doi.org/10.1017/ash.2026.10815.

## References

[1] Said M , van Hougenhouck-Tulleken W , Naidoo R , Mbelle N , Ismail F. Outbreak of *Ralstonia mannitolilytica* bacteraemia in patients undergoing haemodialysis at a tertiary hospital in Pretoria, South Africa. Antimicrob Resist Infect Control 2020;9:117. 10.1186/s13756-020-00778-7 PMC7389438 32727576

[2] Saunders M , Weaver A , Stretch R , et al. Outbreak of *Ralstonia pickettii* associated with contamination of saline products distributed internationally, the United Kingdom, 2024. Euro Surveill 2024;29:2400384. 10.2807/1560-7917.ES.2024.29.27.2400384 PMC11225263 38967014

[3] Menekşe S , Hacıseyitoğlu D , Yıldız SS , Bayrakdar F An outbreak of *Ralstonia pickettii* bloodstream infection and clinical outcomes. J Infect Dev Ctries 2022;16:705–711. 10.3855/jidc.15159 35544634

[4] Demirdag TB , Ozkaya-Parlakay A , Bayrakdar F , et al. An outbreak of *Ralstonia pickettii* bloodstream infection among pediatric leukemia patients. J Microbiol Immunol Infect 2022;55:80–85. 10.1016/j.jmii.2020.12.004 33461864

[5] Çelen SS , Zhumatayev S , Yalçın K , et al. Central line–associated bloodstream infection outbreak related to Ralstonia pickettii–contaminated saline in a pediatric hematopoietic stem cell transplant center. Turk J Pediatr 2024;66:421–428. 10.24953/turkjpediatr.2024.4529 39387430

[6] Alshuhri S , Alosaimi A , Alnafee K , et al. *Ralstonia mannitolilytica* infection in a tertiary care center: an outbreak report. Am J Infect Control 2024;52:1422–1425. 10.1016/j.ajic.2024.09.019 39343302

[7] Tüzemen NU , Önal U , Kazak E , et al. An outbreak of *Ralstonia insidiosa* bloodstream infections caused by contaminated heparinized syringes. J Infect Chemother 2022;28:1387–1392. 10.1016/j.jiac.2022.06.011 35760221

[8] Jhung MA , Sunenshine RH , Noble-Wang J , et al. A national outbreak of *Ralstonia mannitolilytica* associated with use of a contaminated oxygen-delivery device among pediatric patients. Pediatrics 2007;119:1061–1068. 10.1542/peds.2006-3739 17545371

[9] Shankar M , Rampure S , Siddini V , Ballal HS. Outbreak of *Ralstonia mannitolilytica* in hemodialysis unit: a case series. Indian J Nephrol 2018;28:323–326. 10.4103/ijn.IJN_77_17 PMC6094832 30158756

[10] Gröbner S , Heeg P , Autenrieth IB , Schulte B. Monoclonal outbreak of catheter-related bacteraemia by *Ralstonia mannitolilytica* on two haemato-oncology wards. J Infect 2007;55:539–544. 10.1016/j.jinf.2007.07.021 17881058

[11] Fabricci M , Trinca A , Talotti L , et al. A urokinase-associated outbreak of *Ralstonia mannitolilytica* bloodstream infections in haemodialysis patients in north-eastern Italy, January to April 2023. Euro Surveill 2023;28:2300328. 10.2807/1560-7917.ES.2023.28.28.2300328 PMC10347894 37440346

[12] Ryan MP , Adley CC. The antibiotic susceptibility of water-based bacteria *Ralstonia pickettii* and *Ralstonia insidiosa* . J Med Microbiol. 2013;62:1025–1031. 10.1099/jmm.0.054759-0 23579396

[13] Bhatnagar N , Vasanth S , Tejan N , Sahu C. An outbreak of *Ralstonia mannitolilytica* septicaemia at a tertiary care hospital: an observational cross-sectional study. J Clin Diagn Res 2024;18:

[14] Kim G , Yoo RN , So H , et al. Clinical manifestations of *Ralstonia mannitolilytica* infection in pediatric patients and epidemiological investigation of outbreaks. J Korean Med Sci 2023;38:e252. 10.3346/jkms.2023.38.e252 PMC10442498 37605494

[15] Viana-Cardenas E , Triana A , Cardenas-Alvarez J , et al. A large multicenter *Ralstonia pickettii* outbreak in critically ill patients during the COVID-19 pandemic: epidemiological and clinical characteristics of 66 cases. J Infect Prev 2024;25:85–88. 10.1177/17571774241236250 PMC10998545 38584711

[16] Simon L , Ughetto E , Gaudart A , Degand N , Lotte R , Ruimy R. Direct identification of 80% of bacteria from blood culture bottles by matrix-assisted laser desorption ionization–time-of-flight mass spectrometry using a 10-minute extraction protocol. J Clin Microbiol, 2019;57:e01278–e01218. 10.1128/JCM.01278-18 PMC6355546 30463897

[17] Clinical and Laboratory Standards Institute. Performance Standards for Antimicrobial Susceptibility Testing, 34th edition. Wayne, PA: CLSI; 2024.

[18] Centers for Disease Control and Prevention. Standard Operating Procedure for PulseNet PFGE of Escherichia coli O157:H7, Escherichia coli non-O157 (STEC), Salmonella serotypes, Shigella sonnei and Shigella flexneri. Atlanta: CDC. 2013. Code: PNL05.

[19] Liu CX , Yan C , Zhang P , Li FQ , Yang JH , Li XY. Ralstonia mannitolilytica–induced septicemia and homology analysis in infected patients: 3 case reports. Jundishapur J Microbiol 2016;9:e34373. 10.5812/jjm.34373 PMC5035395 27679705

[20] Ryan MP , Adley CC. *Ralstonia* spp.: emerging global opportunistic pathogens. Eur J Clin Microbiol Infect Dis 2014;33:291–304. 10.1007/s10096-013-1975-9 24057141

[21] Anderson RR , Warnick P , Schreckenberger PC. Recurrent CDC group IVc-2 bacteremia in a human with AIDS. J Clin Microbiol 1997;35:780–782. 10.1128/jcm.35.3.780-782.1997 PMC229673 9041435

[22] Nurjadi D , Boutin S , Schmidt K , Ahmels M , Hasche D. Identification and elimination of the clinically relevant multi-resistant environmental bacteria *Ralstonia insidiosa* in primary cell culture. Microorganisms 2020;8:1599. 10.3390/microorganisms8101599 PMC7603027 33080836

