# Supplementary material for: Rapid containment of a hospital outbreak of Ralstonia mannitolilytica bloodstream infection linked to contaminated pre-filled 0.9% saline vials
Source: Antimicrob Steward Healthc Epidemiol. 2026 Sep 1;6(1):e244. doi: 10.1017/ash.2026.10815 (PMC13760306; doi:10.1017/ash.2026.10815)

Supplementary Figure S1.a

Click here to access/download;Figure;Gram film from blood culture.tiff

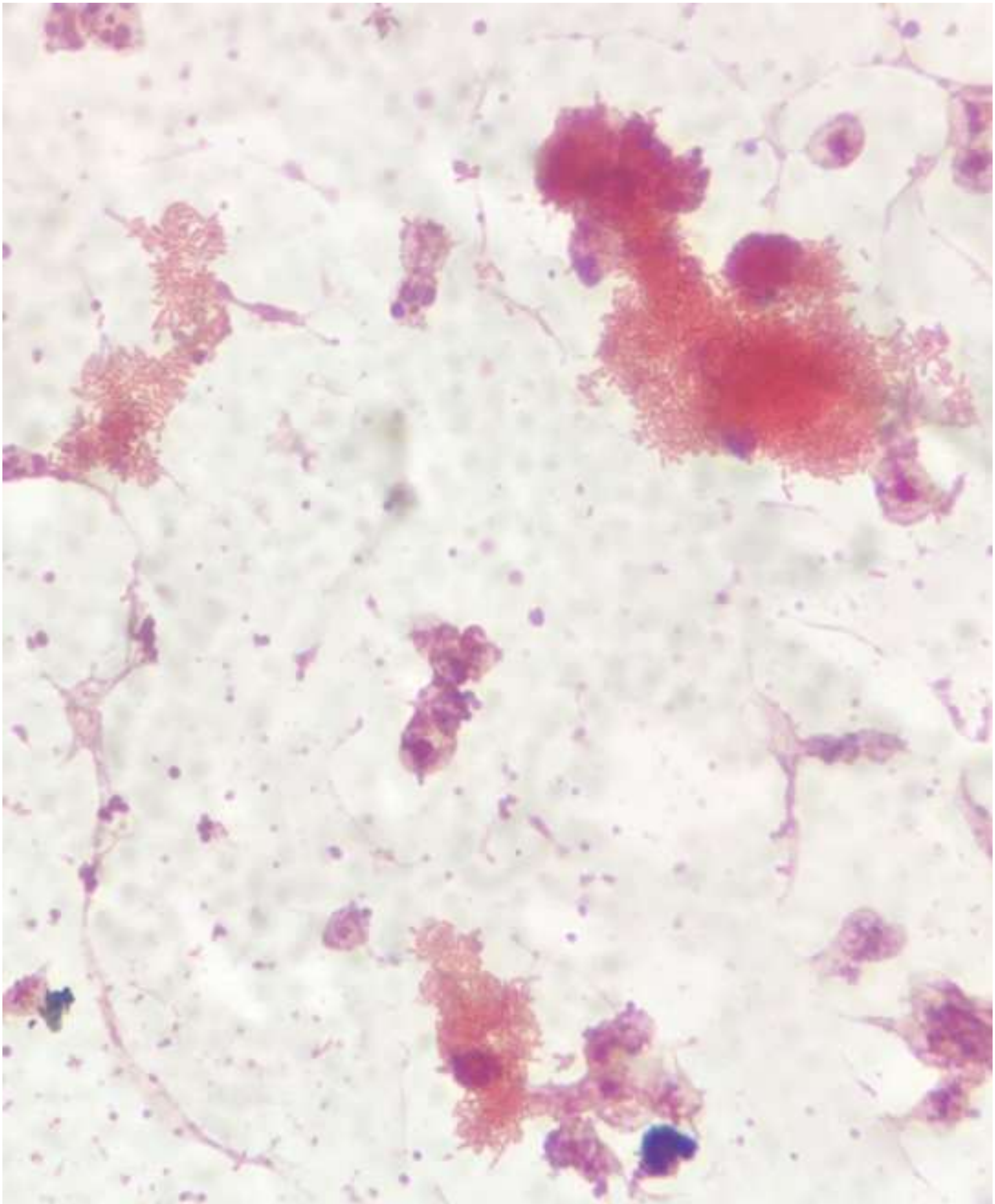

Supplement: Supplementary file 1 [file S2732494X26108158sup001.pdf]
