# Supplementary material for: Rapid containment of a hospital outbreak of Ralstonia mannitolilytica bloodstream infection linked to contaminated pre-filled 0.9% saline vials
Source: Antimicrob Steward Healthc Epidemiol. 2026 Sep 1;6(1):e244. doi: 10.1017/ash.2026.10815 (PMC13760306; doi:10.1017/ash.2026.10815)
Supplement: Supplementary file 5 [file S2732494X26108158sup005.docx]

**SUPPLEMENTARY MATERIAL**

**Supplementary Methods (Pulsed-field gel electrophoresis).** DNA was restricted with SpeI at 37 °C for 2 hours, embedded in 1% casting agarose, and resolved on a CHEF-Mapper system (Bio-Rad) with an initial switch time of 5 s, a final switch time of 35 s, 6 V/cm, and a 120° included angle for 19 hours.19 *Salmonella Braenderup* H9812 served as the molecular size marker. Gels were stained with GelRed, imaged on a Bio-Rad GelDoc system, and band patterns were compared using BioNumerics version 5.1.

**Supplementary Figure S1.** Microbiological appearance of *Ralstonia* isolated from blood cultures (former main-text Figure 2). (a) Gram stain of positive blood culture broth; (b) growth on chocolate agar at 48 hours; (c) growth on CLED agar at 48 hours showing non-lactose-fermenting colonies. Provided as a separate image file.

**Supplementary Figure S2.** PFGE fingerprints of 19 *Ralstonia mannitolilytica* isolates (18 clinical and 1 product; former main-text Figure 3); the dendrogram demonstrates 100% band-pattern similarity, consistent with a single outbreak clone. Provided as a separate image file.

Supplementary Table S1 (former Table 1) follows on the next page in landscape orientation.

**Supplementary Table S1.** Demographic, microbiological, and clinical characteristics of 21 patients with *Ralstonia* bloodstream infection during the outbreak, Sultan Qaboos University Hospital, Oman, 29 September–8 October 2024.

| **Pt #** | **Age / Sex** | **Department** | **Vascular device** | **Isolate(s)** | **Underlying diagnosis** | **Empirical → definitive treatment** | **Outcome / complications** |
| --- | --- | --- | --- | --- | --- | --- | --- |
| 1 | 67 y / M | Adult ICU | CVC | *R. mannitolilytica* | Parkinson's disease; intracerebral hemorrhage | Empiric: piperacillin–tazobactam → Definitive: TMP/SMX × 7 d; CVC removed | None |
| 2 | 5 y / F | Pediatric Hematology | CVC | *R. mannitolilytica* | Acute myeloid leukemia (AML) | Empiric: piperacillin–tazobactam + amikacin → Definitive: TMP/SMX × 14 d; CVC removed | Brief ICU admission for sepsis; complete recovery |
| 3 | 23 y / M | Adult Hematology | Port-a-cath | *R. mannitolilytica* + *R. pickettii* | Sickle cell disease | Empiric: piperacillin–tazobactam → Definitive: TMP/SMX × 7 d; CVC removed | None |
| 4 | 7 y / M | Pediatric Hematology | Peripheral line | *R. mannitolilytica* | AML | Empiric: cefepime + amikacin + TMP/SMX → Definitive: TMP/SMX × 10 d | None |
| 5 | 10 y / F | Pediatric Hematology | Port-a-cath | *R. mannitolilytica* | AML | Empiric: vancomycin + piperacillin–tazobactam + amikacin → Definitive: TMP/SMX × 14 d; device retained | None |
| 6 | 3 y / M | Pediatric Hematology | Port-a-cath | *R. mannitolilytica* | Acute lymphoblastic leukemia (ALL) | Empiric: piperacillin–tazobactam + TMP/SMX → Definitive: TMP/SMX × 14 d; device retained | None |
| 7 | 33 y / M | Adult Hematology | CVC | *R. mannitolilytica* + *R. pickettii* | — | — | None |
| 8 | 4 y / M | Pediatric Hematology | Port-a-cath | *R. mannitolilytica* | ALL | Empiric: vancomycin + piperacillin–tazobactam + TMP/SMX → Definitive: TMP/SMX × 14 d; device retained | None |
| 9 | 10 y / M | Pediatric Hematology | Hickman | *R. mannitolilytica* | Post-HSCT (thalassemia major) | Empiric: piperacillin–tazobactam + TMP/SMX → Definitive: TMP/SMX × 14 d; device retained | None |
| 10 | 6 mo / F | Pediatric ICU | CVC | *R. mannitolilytica* + *R. pickettii* | Pulmonary alveolar proteinosis | Empiric: piperacillin–tazobactam + amikacin → Definitive: TMP/SMX × 14 d; CVC removed | None |
| 11 | 9 y / M | Pediatric Hematology | CVC | *R. mannitolilytica* | ALL | Empiric: piperacillin–tazobactam → Definitive: TMP/SMX × 14 d; line initially retained | Recurrence at 1 wk → CVC removed; second TMP/SMX course × 14 d |
| 12 | 30 y / F | Adult ICU | Peripheral line | *R. mannitolilytica* | Lennox-Gastaut syndrome; pre-eclampsia | Definitive: TMP/SMX × 7 d | None |
| 13 | 6 y / M | Pediatric Hematology | Hickman | *R. mannitolilytica* | Post-HSCT (aplastic anemia) | Empiric: piperacillin–tazobactam → Definitive: TMP/SMX × 14 d; Hickman removed | No vegetations on TTE |
| 14 | 5 y / F | Pediatric Hematology | CVC | *R. mannitolilytica* | Post-HSCT (ALL) | Empiric: piperacillin–tazobactam + amikacin → Definitive: TMP/SMX × 14 d; CVC removed | Brief ICU admission for sepsis |
| 15 | 1 y / F | Pediatric Hematology | Peripheral line / Hickman | *R. mannitolilytica* | Relapsed B-ALL | Empiric: piperacillin–tazobactam + amikacin → Definitive: TMP/SMX × 14 d; Hickman removed | Septic shock; ICU 5 d (NIV, inotropes) |
| 16 | 17 y / M | Adult Hematology | Hickman | *R. mannitolilytica* | Post-HSCT (mixed phenotype acute leukemia) | Empiric: meropenem → Definitive: TMP/SMX × 14 d; CVC removed | None |
| 17 | 5 y / F | Pediatric Hematology | Port-a-cath | *R. mannitolilytica* | ALL | Empiric: piperacillin–tazobactam → Definitive: TMP/SMX × 14 d; device initially retained | Recurrence at 5 wk → device removed; TMP/SMX then levofloxacin |
| 18 | 19 y / M | Adult Hematology | PICC | *R. mannitolilytica* | Post-HSCT (ALL) | Empiric: piperacillin–tazobactam → Definitive: TMP/SMX × 7 d; PICC removed | None |
| 19 | 44 y / M | Adult Hematology | PICC | *R. mannitolilytica* | Post-HSCT (chronic myeloid leukemia) | Empiric: piperacillin–tazobactam → Definitive: TMP/SMX × 7 d; PICC removed | None |
| 20 | 30 y / M | Adult Hematology | PICC | *R. mannitolilytica* | Post-HSCT (B-ALL) | Empiric: piperacillin–tazobactam → Definitive: TMP/SMX × 7 d; PICC removed | None |
| 21 | 12 y / F | Pediatric Hematology | Port-a-cath | *R. mannitolilytica* + *R. pickettii* | B-ALL (Regimen C) | Definitive: TMP/SMX × 14 d + ceftazidime lock therapy × 10–14 d; line retained | None |

Abbreviations: y, years; mo, months; M, male; F, female; ICU, intensive care unit; CVC, central venous catheter; PICC, peripherally inserted central catheter; HSCT, hematopoietic stem-cell transplantation; AML, acute myeloid leukemia; ALL, acute lymphoblastic leukemia; B-ALL, B-cell acute lymphoblastic leukemia; TMP/SMX, trimethoprim–sulfamethoxazole; NIV, non-invasive ventilation; TTE, transthoracic echocardiography; “—”, not available.
